# External Validation Of The Updated ADO Score In COPD Patients From The Birmingham COPD Cohort

**DOI:** 10.2147/COPD.S212381

**Published:** 2019-10-24

**Authors:** Spencer J Keene, Rachel E Jordan, Frits ME Franssen, Frank de Vries, James Martin, Alice Sitch, Alice Margaret Turner, Andrew P Dickens, David Fitzmaurice, Peymane Adab

**Affiliations:** 1 Institute of Applied Health Research, College of Medical and Dental Sciences, University of Birmingham, Birmingham, UK; 2 Department of Clinical Pharmacy & Toxicology, Maastricht University Medical Center+, Maastricht, the Netherlands; 3 Ciro, Horn, the Netherlands; 4 Department of Respiratory Medicine, NUTRIM School of Nutrition and Translational Research in Metabolism, Maastricht University Medical Centre+, Maastricht, The Netherlands; 5 Utrecht Institute for Pharmaceutical Sciences Utrecht University, Utrecht, The Netherlands; 6 NIHR Birmingham Biomedical Research Centre, University Hospitals Birmingham NHS Foundation Trust and University of Birmingham, Birmingham, UK; 7 Clinical Trials Unit, Warwick Medical School, University of Warwick, Coventry, UK

**Keywords:** pulmonary disease, chronic obstructive, mortality, prognosis, validation studies

## Abstract

**Background:**

Reviews suggest that the ADO score is the most discriminatory prognostic score for predicting mortality among chronic obstructive pulmonary disease (COPD) patients, but a full evaluation and external validation within primary care settings is critical before implementation.

**Objectives:**

To validate the ADO score in prevalent and screen-detected primary care COPD cases at 3 years and at shorter time periods.

**Patients and methods:**

One thousand eight hundred and ninety-two COPD cases were recruited between 2012 and 2014 from 71 United Kingdom general practices as part of the Birmingham COPD Cohort study. Cases were either on the practice COPD register or screen-detected. We validated the ADO score for predicting 3-year mortality with 1-year and 2-year mortality as secondary endpoints using discrimination (area-under-the-curve (AUC)) and calibration plots.

**Results:**

One hundred and fifty-four deaths occurred within 3 years. The ADO score was discriminatory for predicting 3-year mortality (AUC= 0.74; 95% CI: 0.69–0.79). Similar performance was found for 1- (AUC= 0.73; 0.66–0.80) and 2-year mortality (0.72; 0.67–0.76). The ADO score showed reasonable calibration for predicting 3-year mortality (calibration slope 0.95; 0.70–1.19) but over-predicted in cases with higher predicted risks of mortality at 1 (0.79; 0.45–1.13) and 2-year (0.79; 0.57–1.01) mortality.

**Discussion:**

The ADO score showed promising discrimination in predicting 3-year mortality in a primary care population including screen-detected cases. It may need to be recalibrated if it is used to provide risk predictions for 1- or 2-year mortality since, in these time-periods, over-prediction was evident, especially in cases with higher predicted mortality risks.

## Plain Language Summary

Prediction models are tools that can be used to provide estimates of likely outcomes, such as death, over a specified time period in individual patients. This information can then be used to inform treatment decisions. For example, the intensity of treatment (or monitoring) may be increased for those with higher individual risks. These tools are usually developed using data from one group of people. However, because other groups of people may have different characteristics, the accuracy of the tool needs to be checked in these other groups. The ADO (age, dyspnoea (i.e. breathlessness), and obstructed airways) score was developed to predict death within 3 years in people with COPD. Our aim was to check whether the ADO score is accurate in predicting the risk of death in a group of people with COPD identified in general practices in the UK. We also wanted to determine whether it was accurate for predicting the risk of death at time periods shorter than 3 years. Previous studies have shown that the ADO score distinguishes well between likelihood of being dead or alive (i.e. the discrimination of a model). In our sample of people with newly diagnosed and existing COPD in primary care, we confirmed these results. However, previous studies have not properly assessed the degree of agreement between the expected and observed individual risk of death (i.e. the calibration of a model). It is essential to report calibration in prognostic models because it tells you how accurate mortality predictions are likely to be for individual with a particular disease. We found that the ADO score over-predicts individual risk of death for periods <3 years. Unless adjusted, this reduces its usefulness for clinical decision-making. In addition, this has implications for other COPD prognostic scores that have been tested and used at shorter time periods than they were developed for.

## Introduction

Chronic obstructive pulmonary disease (COPD) is the third leading cause of mortality worldwide.^1^,^2^ Prognostic scores to predict mortality risk in people with COPD are useful in order to assess disease severity, define intervention options, and facilitate consultations with patients about their prognosis.^3^ Knowledge of the risk of mortality also allows the benefits of treatments for COPD to be weighed against potential harms, such as side effects, costs, and inconvenience^2^ in order to enable informed clinical decision-making. The extent of airflow obstruction, usually assessed by forced expiratory volume in the first second (FEV_1_), has long been recognised as an important measure of prognosis and is used for disease staging.^2^ However, the complex and multifaceted nature of COPD^4^,^5^ has led to the identification of other important predictors of mortality and the recognition that combining these in multicomponent indices^6^–^10^ improves prognostic ability. However, before implementation in clinical practice, it is important to evaluate the predictive ability of the prognostic index in different populations.^3^ There are two important aspects to such evaluation, including assessment of how well the index can differentiate between those who die and those who remain alive (i.e. discrimination) and the extent of agreement between predicted and observed mortality (i.e. calibration). The latter is particularly important for prognostication.^11^

Amongst prognostic indices, the ADO (age, dyspnoea, airflow obstruction) score has wide applicability as it is made up of only three easily measured components,^9^ overcoming the limitation of many other indices.^12^ The original ADO score was developed in 2009^9^ to predict 3-year mortality in patients with moderate-to-severe COPD from secondary care and was updated in 2012 in an international cohort from a variety of healthcare settings to improve its generalisability.^13^ The updated ADO has been externally validated several times.^13^–^16^ However, only two validation studies were in primary care populations,^14^,^16^ where most people with COPD are cared for.^17^ In one of these studies, calibration was not assessed.^14^ The other study only considered 2-year mortality as the outcome and adjusted the intercept of the ADO score.^16^ A further two studies used populations across primary, secondary and tertiary settings.^13^,^15^ However, no analyses were undertaken to assess the differential performance of the ADO score in each setting.

Our aim was to validate the updated ADO score in COPD cases from a large primary care research cohort (the Birmingham COPD cohort) which included both previously and newly diagnosed cases and where dyspnoea and lung function were measured under standardized conditions.

## Methods

This paper was written in accordance with the Transparent Reporting of a multivariable prediction model for Individual Prognosis Or Diagnosis statement.^18^

### Design

External validation study of a published prognostic score.

### Source And Study Population

The characteristics of the Birmingham COPD cohort, which is part of the Birmingham Lung Improvement Studies (BLISS), have been summarized in a previous publication.^19^ Briefly, COPD cases were recruited from 71 UK general practices across the West Midlands, United Kingdom. For this analysis, cohort cases with diagnosed COPD (aged 40 and over) on practice Quality and Outcomes Framework COPD registers (i.e. prevalent cases) and those with newly detected COPD identified through a case-finding trial (i.e. incident cases were screen-detected)^20^ were included. The definition of COPD in incident cases was based on reporting of relevant symptoms in those with airflow obstruction (forced expiratory volume in the first second (FEV_1_)/forced vital capacity (FVC) <0.7 according to recommendations in UK guidelines). Baseline assessments took place at cohort entry from 31 May 2012 to 25 June 2014.

### Exposure And Outcome Measurements

The ADO score (0–14) was computed from three variables taken at baseline: age, dyspnoea (modified MRC score), and obstruction (FEV_1_% predicted). Age was calculated from patient-reported date of birth, and dyspnoea was assessed by a questionnaire using the British Medical Research Council guidelines.^21^ A researcher trained to international standards to measure FEV_1_ administered the nddEasy One Spirometer (ndd, Switzerland) before (max eight blows) and after (max six blows) 400µg salbutamol, aiming for three blows within 100 mL. FEV_1_ and FVC recording were considered useable if they met ATS acceptability criteria and were within 200 mL. The highest recording was then taken.^19^ Quality assurance was maintained using real-time quality assessment, with over-reading of spirometry measurements. FEV_1_% predicted was estimated using the Global Lung Function Initiative equations.^22^

Linked mortality data were obtained through the Office of National Statistics for the period of recruitment until 31 March 2016 through NHS Digital.^23^ Other patient characteristics including ethnicity, level of deprivation (using Index of Multiple Deprivation derived from home postcode), smoking status, quality of life, and medical history (including self-reported comorbidities and previous exacerbations) were obtained by patient self-report through standardized questionnaires. Body mass index (BMI derived from height and weight measurements) and exercise capacity (using sit-to-stand test) were obtained by trained researchers using standardised protocols at the baseline visit.^19^

### Patient Selection Criteria

The ADO score was developed for participants 40 years and older. Missing baseline mMRC scores or FEV_1_% predicted observations were imputed using multiple imputation (MI) by chained equations so that all remaining incident and prevalent cases (N= 1892) could be included in the final analyses (baseline tables show data prior to imputation). Additional auxiliary variables (cardiovascular disease history, cardiovascular disease medication, chronic cough, chronic phlegm, ethnicity, and gender) were used to aid the imputation. The number of imputed datasets used was based on the fraction of missing data for all variables (11%, so 11 MI datasets were used).^24^ Death data were complete for all cases under the assumption that patients without a date of death remained alive.

### Analysis

Baseline characteristics were compared between prevalent and incident cases as well as between those who died within 3 years of study entry compared to those who did not. Chi-square and Student’s *t*-tests were used for categorical and continuous variables, respectively.

The updated ADO score regression coefficients and intercept^13^ were used to compute the predicted probability of 3-year mortality for each eligible cohort participant (Supplementary Table 1). To assess discrimination, area-under-the-curve (AUC) was estimated with a 95% confidence interval (95% CI) and plotted using AUC-ROC plots.^25^ Calibration was assessed by comparing the predicted probability to the observed probability of mortality and examined with a calibration plot and calibration slope with 95% CI. Calibration plots (STATA function: *pmcalplot*) displayed observed risk by deciles of the predicted risk and also examined risk at the individual level using Locally Weighted Scatterplot Smoothing algorithms.^26^ An estimate of the Calibration-in-the-large (CITL) was used to indicate whether the predictions were systematically too high or too low.^26^ As MI datasets were used, the AUC and calibration slope were estimated in each individual MI dataset, before Rubin’s rule was used to combine estimates.^27^

A Kaplan–Meier plot was created according to ADO score group (0 to 5, 6 and 7, 8 and 9, and 10 to 14). Scores were grouped based on the number of patients. Separation of Kaplan–Meier curves for ADO score groups indicates better discriminative performance.

In secondary analyses (using the same discrimination and calibration methods as above), we evaluated the ability of the ADO index to predict mortality at 1 and 2 years. The period end dates for each case were 1, 2, and 3 years after study entry. If the end date for the period fell after the 31 March 2016, then the case was excluded from that period. Period exclusions were made regardless of whether and at what time cases died to ensure that dead and alive cases were treated the same. However, a sensitivity analysis was performed by re-introducing cases that died within a certain period despite a period end date that fell after the 31 March 2016. Two additional sensitivity analyses were conducted: 1) We estimated the discrimination and calibration estimates for prevalent cases alone and 2) for complete cases (non-missing obstruction and dyspnoea). Prevalent cases were studied alone because the accuracy of the ADO score may be affected by the inclusion of screen-detected cases (which might not reflect usual primary care populations). All analyses were undertaken using STATA (StataCorp, College Station TX, USA).

## Results

**Figure 1:**
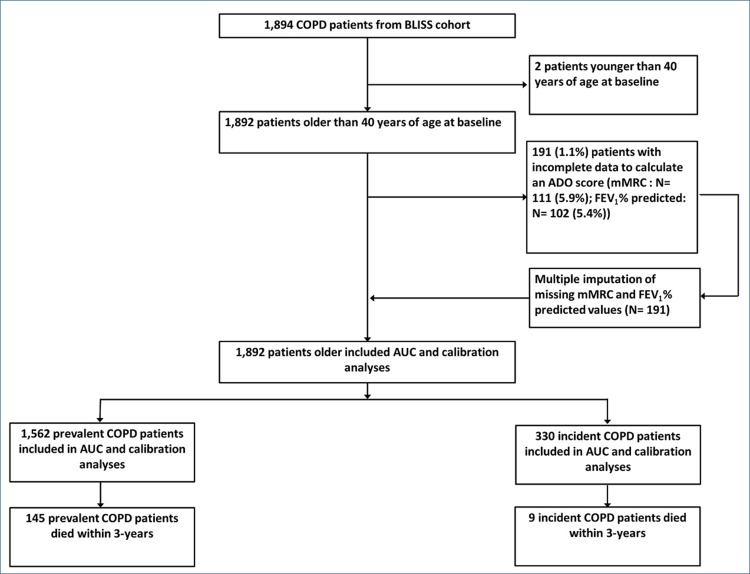
Patient (i.e. case) flow chart from whole cohort to ADO validation sample.

Out of 1894 cases in the cohort, two were younger than 40 years of age at baseline, 111 (5.9%) had missing mMRC score, and 102 (5.4%) had missing FEV_1_% predicted values (22 (1.2%)) were missing both (Figure 1). Before imputing missing mMRC and FEV_1_% predicted, there were 1392 prevalent and 309 incident cases (total 1701). The median observation time was 3.02 years (minimum 1.77 and maximum 3.83 years). The average age was 68.4 years old and 651 (38.3%) cases were female. The majority (79.5%) had mild-to-moderate airflow obstruction (50.6% with GOLD stage II) and the mean ADO score at baseline was 7.0 (SD 2.4). One hundred and twenty-four (7.3%) deaths occurred within 3 years of observation time, 116 (94%) of which occurred in the prevalent cases.

When compared to incident cases, prevalent cases tended to have a worse baseline ADO score (older age, more severe obstruction and worse breathlessness), lower exercise capacity scores, more comorbidities, were more likely to report a worse health-related quality of life score, had more former smokers, and were more likely to report a respiratory hospitalisation and at least one exacerbation in the previous 12 months (Table 1).

**Table 1:** Comparison Of Baseline Characteristics Of Existing COPD Cases With Those Who Were Screen Detected (N=1701)

	Prevalent Cases N= 1392	Incident Cases N= 309	Total N= 1701	P-Value^a^
Female – N (%)	528 (37.9)	123 (39.8)	651 (38.3)	0.540
Age in years – N (%)				
40–49	46 (3.3)	18 (5.8)	64 (3.8)	**<0.001**
50–59	167 (12.0)	65 (21.0)	232 (13.6)	
60–69	536 (38.5)	119 (38.5)	655 (38.5)	
70–79	469 (33.7)	106 (34.3)	575 (33.8)	
80+	174 (12.5)	1 (0.3)	175 (10.3)
GOLD^b^ – N (%)				
Mild (FEV_1_≥ 80% of normal)	317 (22.8)	175 (56.6)	492 (28.9)	**<0.001**
Moderate (FEV_1_ ≥ 50 & <80% of normal)	734 (52.7)	127 (41.1)	861 (50.6)	
Severe (FEV_1_ ≥ 30 & <50% of normal)	285 (20.5)	6 (1.9)	291 (17.1)	
Very severe (FEV_1_ ≥ 0 & <30% of normal)	56 (4.0)	1 (0.3)	57 (3.4)
FEV_1_% predicted – mean(SD)	64.61 (20.3)	82.51 (16.6)	67.80 (20.8)	**<0.001**
FEV_1_/FVC ratio – mean(SD)	0.55 (0.1)	0.63 (0.1)	0.57 (0.1)	**<0.001**
mMRC dyspnoea – N (%)				
0	233 (16.7)	106 (34.3)	339 (19.9)	**<0.001**
1	301 (21.6)	96 (31.1)	397 (23.3)	
2	307 (22.1)	62 (20.1)	369 (21.7)	
3	244 (17.5)	22 (7.1)	266 (15.6)	
4	307 (22.1)	23 (7.4)	330 (19.4)
Baseline ADO – mean(SD)	7.41 (2.4)	5.20 (1.9)		**<0.001**
Baseline ADO groups – N (%)				
0 to 5	265 (19.0)	170 (55.0)	435 (25.6)	**<0.001**
6 to 7	471 (33.8)	101 (32.7)	572 (33.6)	
8 to 9	392 (28.2)	37 (12.0)	429 (25.2)	
10 to 14	264 (19.0)	1 (0.3)	265 (15.6)
White British/mixed British - N (%)	1,181 (84.8)	260 (84.1)	1441 (84.7)	0.757
Missing	98 (7.0)	23 (7.4)		
IMD^c^ deprivation score – N (%)				**0.031**
Most deprived – Quintile 1	290 (20.8)	49 (15.9)	339 (20.2)	
Quintile 2	265 (19.0)	68 (22.0)	333 (19.8)	
Quintile 3	249 (17.9)	71 (23.0)	320 (19.0)	
Quintile 4	292 (21.0)	60 (19.4)	352 (20.9)	
Least deprived – Quintile 5	288 (20.7)	50 (16.2)	338 (20.1)
Missing	8 (0.6)	11 (3.6)		
Exercise capacity^d^ – N (%)				
Worst – 0 to 9	73 (5.2)	12 (3.9)	85 (6.1)	**<0.001**
10 to 19	618 (44.4)	85 (27.5)	703 (50.0)	
20 to 29	418 (30.0)	136 (44.0)	554 (39.4)	
30 to 39	33 (2.4)	23 (7.4)	56 (4.0)	
Best – 40 to 50	5 (0.4)	3 (1.0)	8 (0.6)
Missing	245 (17.6)	50 (16.18)		
BMI groups – N (%)				
0–18.49	29 (2.1)	3 (1.0)	32 (2.0)	0.340
18.50–24.99	338 (24.3)	62 (20.1)	400 (24.8)	
25.00–29.99	522 (37.5)	105 (34.0)	627 (38.9)	
30.00+	447 (32.1)	104 (34.0)	551 (34.2)
Missing	56 (4.0)	35 (11.3)		
Smoking group – N (%)				
Never smoker	130 (9.3)	43 (13.9)	173 (10.9)	**0.005**
Current smoker	369 (26.5)	95 (30.7)	464 (29.4)	
Former smoker	795 (57.1)	149 (48.2)	944 (59.7)
Missing	98 (7.0)	22 (7.1)		
HRQL^e^ category – *N* (%)				
Low impact – 0 to 9	139 (10.0)	71 (23.0)	210 (16.4)	**<0.001**
10 to 19	374 (26.9)	105 (34.0)	479 (37.4)	
20 to 29	381 (27.4)	51 (16.5)	432 (33.7)	
Severe impact – 30 to 40	153 (11.0)	8 (2.6)	161 (12.6)
Missing	345 (24.8)	74 (24.0)		
Exacerbation in last 12 months – N (%)	820 (58.9)	78 (25.2)	898 (54.5)	**<0.001**
Missing	44 (3.2)	9 (2.9)		
Cardiovascular disease history – N (%)	776 (55.8)	135 (43.7)	911 (53.6)	**<0.001**
Any cancer – N (%)	173 (12.4)	39 (12.6)	212 (13.9)	0.737
Missing	162 (11.6)	16 (5.2)		
Asthma – N (%)	565 (40.6)	84 (27.2)	649 (42.6)	**<0.001**
Missing	155 (11.1)	21 (6.8)		
Osteoporosis – N (%)	104 (7.8)	23 (7.4)	127 (8.8)	0.636
Missing	239 (17.2)	26 (8.4)		
Depression – N (%)	255 (18.3)	68 (22.0)	323 (21.7)	0.360
Missing	190 (13.7)	22 (7.1)		
Respiratory hospital admission in previous 12 months – N (%)	82 (5.9)	3 (1.0)	85 (5.0)	**<0.001**

**Notes:** Missing rows were added only for variables with missing data. Bold denotes statistical significance. ^a^*P*-values describe differences in characteristics between cohorts without accounting for missing as a separate category. Chi-square test for categorical data and Student’s *t*-test for continuous data. ^b^The Global Initiative for Chronic Obstructive Lung Disease (GOLD) categories of airflow limitation.^c^Based on the Index of Multiple Deprivation (IMD) 2010. Lower quintiles indicate more deprivation. ^d^Assessed using the sit-to-stand test.^28^ eHealth-related quality of life based on the COPD Assessment Test (CAT). **Abbreviations:** FEV_1_, forced expiratory volume in one second; MRC, medical research council; HRQL, health-related quality of life.

Table 2 shows a comparison of characteristics of cases according to whether or not they were alive within 3 years of observation time. Those who died were older and had more severe obstruction and dyspnoea (all *P*<0.001) which resulted in a higher baseline ADO score (mean (SD) score 8.98 (2.14)) compared to those who remained alive (6.85 (2.39)). They were also less likely to be female, had poorer exercise capacity, lower BMI, were more likely to have a severe impact of COPD on health-related quality of life, were more likely to have cardiovascular comorbidity, and were more likely to report respiratory hospitalisation in the 12 months before baseline compared to those who remained alive.

**Table 2:** Baseline Characteristics Of Cases (N=1701) By Whether Or Not They Died Within 3 Years Of Observation Time

	Alive Within 3 Years Of Observation Time (N=1577)	Dead Within 3 Years Of Observation Time (N=124)	Total (N= 1701)	P-Value^a^
Female – N (%)	618 (39.2)	33 (26.6)	651 (38.3)	**0.006**
Age in years – N (%)				
40–49	62 (3.9)	2 (1.6)	64 (3.8)	**<0.001**
50–59	227 (14.4)	5 (4.0)	232 (13.6)	
60–69	619 (39.3)	36 (29.0)	655 (38.5)	
70–79	529 (33.5)	46 (37.1)	575 (33.8)	
80+	140 (8.9)	35 (28.2)	169 (9.9)
GOLD^b^ stage – N (%)				
Mild (FEV_1_≥ 80% of normal)	479 (30.4)	13 (10.5)	492 (28.9)	**<0.001**
Moderate (FEV_1_ ≥ 50 & <80% of normal)	803 (50.9)	58 (46.8)	861 (50.6)	
Severe (FEV_1_ ≥ 30 & <50% of normal)	252 (16.0)	39 (31.5)	291 (17.1)	
Very severe (FEV_1_ ≥ 0 & <30% of normal)	43 (2.7)	14 (11.3)	57 (3.4)
FEV_1_% predicted – mean(SD)	69.0 (20.5)	55.2 (20.6)	68.0 (20.8)	**<0.001**
FEV_1_/FVC ratio – mean(SD)	0.57 (0.13)	0.52 (0.15)	0.57 (0.13)	**<0.001**
mMRC dyspnoea – N (%)				
0	321 (20.4)	18 (14.5)	339 (19.9)	**<0.001**
1	382 (24.2)	15 (12.1)	397 (23.3)	
2	343 (21.8)	26 (21.0)	369 (21.7)	
3	243 (15.4)	23 (18.6)	266 (15.6)	
4	288 (18.3)	42 (33.9)	330 (19.4)
Baseline ADO – mean (SD)	6.85 (2.39)	8.98 (2.14)	7.01 (2.43)	**<0.001**
Baseline ADO groups – N (%)				
Low risk – 0 to 5	428 (27.1)	7 (5.7)	435 (25.6)	**<0.001**
6 to 7	548 (34.8)	24 (19.4)	572 (33.6)	
8 to 9	390 (24.7)	39 (31.5)	429 (25.2)	
High risk – 10 to 14	211 (13.4)	54 (43.6)	265 (15.6)
White British/mixed British – N (%)	1331 (84.4)	110 (88.7)	1441 (84.7)	0.425
Missing	115 (7.3)	6 (4.8)	121 (7.1)	
IMD^c^ deprivation score – N (%)				0.406
Most deprived – Quintile 1	316 (20.0)	23 (18.6)	339 (19.9)	
Quintile 2	302 (19.2)	31 (25.0)	333 (19.6)	
Quintile 3	302 (19.2)	18 (14.5)	320 (18.8)	
Quintile 4	324 (20.6)	28 (22.6)	352 (20.7)	
Least deprived – Quintile 5	316 (20.0)	22 (17.7)	338 (19.9)
Missing	17 (1.1)	2 (1.6)	19 (1.1)	
Exercise capacity^d^ – N (%)				
Worst – 0 to 9	75 (4.8)	10 (8.1)	85 (5.0)	**<0.001**
10 to 19	644 (40.8)	59 (47.6)	703 (41.3)	
20 to 29	536 (34.0)	18 (14.5)	554 (32.6)	
30 to 39	55 (3.5)	1 (0.8)	56 (3.3)	
Best – 40 to 50	8 (0.5)	0	8 (0.5)
Missing	259 (16.4)	36 (29.0)	295 (17.3)	
BMI groups - N (%)				
Underweight – 0–18.49 kg/m^2^	25 (1.6)	7 (5.7)	32 (1.9)	**0.003**
Normal – 18.50–24.99 kg/m^2^	365 (23.2)	35 (28.2)	400 (23.5)	
Overweight – 25.00–29.99 kg/m^2^	583 (37.0)	44 (35.5)	627 (36.9)	
Obese – 30.00 + kg/m^2^	520 (33.0)	31 (25.0)	551 (32.4)
* *Missing	84 (5.3)	7 (5.7)	91 (5.4)	
Smoking group – N (%)				
Never smoker	165 (10.5)	8 (6.5)	173 (10.2)	0.359
Current smoker	428 (27.1)	36 (29.0)	464 (27.3)	
Former smoker	873 (55.4)	71 (57.3)	944 (55.5)
Missing	111 (7.0)	9 (7.3)	120 (7.1)	
HRQL^e^ category – N (%)				
Low impact – 0 to 9	191 (12.1)	19 (15.3)	210 (12.4)	**0.006**
10 to 19	450 (28.5)	29 (23.4)	479 (28.2)	
20 to 29	403 (25.6)	29 (23.4)	432 (25.4)	
Severe impact – 30 to 40	138 (8.8)	23 (18.6)	161 (9.5)
* *Missing	395 (25.1)	24 (19.4)	419 (24.6)	
Exacerbation in last 12 months – N (%)	830 (52.6)	68 (54.8)	898 (52.8)	0.619
Missing	49 (3.1)	4 (3.2)	53 (3.1)	
Cardiovascular disease history – N (%)	823 (52.2)	88 (71.0)	911 (53.6)	**<0.001**
Any cancer – N (%)	192 (12.2)	20 (16.1)	212 (12.5)	0.195
Missing	165 (10.5)	13 (10.5)	178 (10.5)	
Asthma – N (%)	611 (38.7)	38 (30.7)	649 (38.2)	0.148
Missing	158 (10.0)	18 (14.5)	176 (10.4)	
Osteoporosis – N (%)	119 (7.6)	8 (6.5)	127 (7.5)	0.689
Missing	244 (15.5)	21 (16.9)	265 (15.6)	
Depression – N (%)	304 (19.3)	19 (15.3)	323 (19.0)	0.283
Missing	196 (12.4)	16 (12.9)	212 (12.5)	
Respiratory hospital admission in previous 12 months – N (%)	66 (4.2)	19 (15.3)	85 (5.0)	**<0.001**

**Notes:** Missing rows were added only for variables with missing data. Bold denotes statistical significance. ^a^P-values describe differences in characteristics between cohorts without accounting for missing as a separate category. Chi-square test for categorical data and Student’s *T*-test for continuous data.^b^ The Global Initiative for Chronic Obstructive Lung Disease (GOLD) categories of airflow limitation. ^c^Based on the Index of Multiple Deprivation (IMD) 2010. Lower quintiles indicate more deprivation. ^d^Assessed using the sit-to-stand test.^28 e^Health-related quality of life based on the COPD Assessment Test (CAT). **Abbreviations:** FEV_1_, forced expiratory volume in one second; MRC, medical research council; HRQL, health-related quality of life.

**Figure 2:**
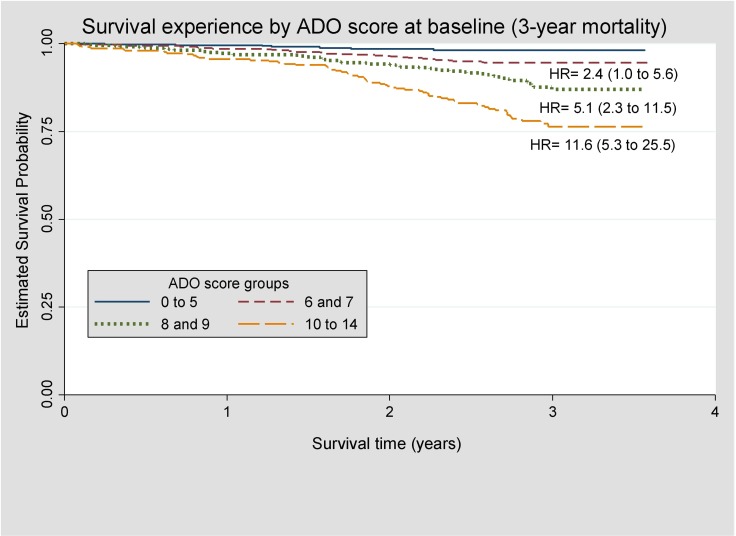
Kaplan–Meier plot of survival experience of patients by ADO score group at baseline. ADO score 0 to 5 used as the reference group (N= 1701).

Figure 2 shows a Kaplan–Meier plot of the survival of cases according to their ADO score at baseline. The survival curves are well separated which indicates good discrimination. Cases with an ADO score of 10 or higher had nearly 12 times the rate of death when compared to patients with an ADO of 0 to 5.

**Figure 3:**
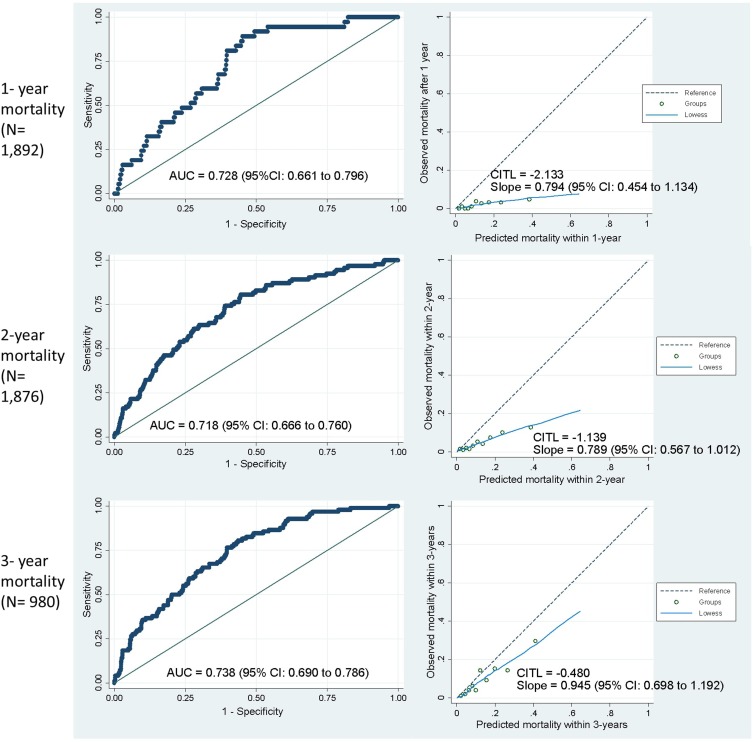
Area-under-the-curve and calibration plots comparing observed and predicted mortality for incident and prevalent cases.

Figure 3 shows AUC and calibration plots for prevalent and incident cases. One thousand eight hundred and ninety-two cases were available after imputing missing mMRC and FEV_1_% predicted observations which added 30 more deaths (total equal to 154 deaths) within 3 years of observation time (Figure 1). For 3-year mortality (N= 980), the ADO score was able to discriminate fairly well between patients who died (N= 98) and those who remained alive (AUC= 0.74; 95% CI: 0.69–0.79). Discriminative ability remained consistent for 1-year (N= 1892, 37 died; AUC=0.73; 95% CI: 0.66–0.80) and 2-year (N= 1,876, 93 died; AUC= 0.72; 95% CI: 0.67–0.76) mortality. Calibration plots showed that the ADO score accurately predicted 3-year mortality (calibration slope= 0.95; 95% CI: 0.70 to 1.19) but over-prediction was evident in those with higher predicted risks of mortality at 1- (0.79; 95% CI: 0.45 to 1.13) and 2-year (0.79; 95% CI: 0.57 to 1.01) time periods. Predictions were also too high (i.e. CITL< 0) at all time-periods; however, these improved as the time periods lengthened. Re-introducing cases that died within a period but with period end dates after the 31 March 2016 only affected the 3-year mortality outcome (N=1,036) and resulted in worse discimination (AUC= 0.71; 95% CI: 0.67-0.76) and calibration (slope= 0.82; 95% CI: 0.62-1.02) (data not shown). An additional sensitivity analysis with only prevalent patients showed similar results for discriminative performance and calibration slopes (Supplementary Table 2). In the complete cases, the calibration slope was decreased to 0.73 at 1-year mortality when compared to the analysis that included all cases. At 3-year mortality, calibration slope increased to 1.08 while discrimination increased to 0.77.

## Discussion

In this external validation study in a primary care COPD population which included screen-detected and prevalent cases, we found that the updated ADO score^13^ was discriminatory with an AUC of 0.74 for predicting 3-year mortality. Discrimination remained stable when predicting 1- and 2-year mortality. However, we found that the ADO score tended to over-predict mortality, especially among the few patients with higher predicted risks of mortality at 1- and 2-year time frames.

Our finding of an AUC of 0.74 is lower than the development model (AUC= 0.85)^13^ but consistent with estimates from two other studies that validated the ADO score for predicting 3-year mortality, one in primary care (AUC= 0.724, 95% CI: 0.719–0.730; mean FEV_1_% predicted of participants: 59.5)^14^ and the other across multiple healthcare settings (AUC= 0.73, 95% CI: 0.70–0.76; FEV_1_% predicted 65.9).^13^ However, a third study used a network meta-analysis to pool data on patients across many healthcare settings and found that the discriminative performance of the ADO score was below 0.70 but still better than nine other prognostic scores.^15^ Our findings are consistent with the results of one primary care study for 1-year (AUC= 0.720; 95% CI: 0.710–0.729) and 2-year (AUC= 0.725; 95% CI: 0.718–0.731) mortality,^14^ but slightly less accurate than a second study for 2-year (AUC= 0.78; 95% CI: 0.71–0.84)^16^ mortality since the upper CI of our 2-year AUC estimate was slightly lower than 0.78.

Accurate calibration is particularly important for evaluating prognostic models because predicted and observed risk need to closely match for predictions to be clinically useful.^11^ This is the first study that reports the calibration slope of the ADO score when predicting 3-year mortality. In addition to 3-year mortality, predictions using shorter time frames are important because clinicians rely on multicomponent prediction models to identify patients nearing the end of life who may benefit from palliative care.^29^ No other studies have assessed calibration for shorter time periods without adjusting the model. We have shown that over-prediction was more pronounced in patients with higher predicted risks of mortality for these time periods. Thus, our findings suggest that recalibration, for example, by using statistical shrinkage techniques,^30^ is needed, in order for the ADO score to better predict mortality over a shorter time frame.

Our study overcomes several limitations found in previous validation studies. For example, we used recommended statistical approaches for predicting mortality in a validation study.^26^ Using a research dataset, such as the Birmingham COPD cohort, had the advantage of more accurate and higher quality measurements at prescribed time points, particularly for spirometry. On the other hand, the Birmingham COPD cohort is not completely representative of all primary care patients with COPD. Ethnic diversity was limited. Additionally, patients needed to be mobile to take part in the cohort study and, therefore, patients with more severe disease who were housebound were more likely to be excluded. Since we used a fixed ratio (based on UK guideline recommendations) instead of a lower limit of normal of FEV1/FVC to define COPD, overdiagnosis may have occurred in older patients.^31^ However, the ADO score was developed in a population where COPD was defined using the fixed ratio^9^ and using the lower limit of normal could lead to underdiagnosis compared to expert opinion.^32^ Furthermore, in a study of 24,207 US adults from 4 cohorts, COPD-related hospitalization and mortality were not significantly different when using the fixed ratio of FEV1/FVC < 0.70 compared to the lower limit of normal to define COPD.^33^ This indicates that our results would not be very different if we had used a lower limit of normal to define our cohort. We included screen-detected COPD cases who, predictably, had very few deaths. However, other studies have not included screen-detected cases despite at least 50% of the COPD population remaining undiagnosed worldwide.^34^ It is important to assess the validity of prognostic indices to predict mortality in this population to inform treatment decisions. Finally, a very small number of deceased patients may have had delayed death registration due to a variety of reasons such as suspicious, unexpected, or accidental deaths.^35^ In addition to the loss of power (i.e. fewer deaths), if patients were considered alive when they were truly dead, then this would result in weaker prognostic accuracy.

## Conclusion

It is well-known that prognostic scores are rarely used in clinical practice for managing people with COPD, especially in primary care.^3^ Although the ADO score is attractive because of its accurate discriminative ability and ease of measurement and calculation in a primary care setting recalibration is needed to improve risk prediction for shorter time frames. Currently, when predicting 1- and 2-year mortality, the ADO score may not be accurate in primary care populations because over-prediction was evident, especially in those with higher predicted risks of mortality and people with COPD may be given treatment that is not needed as a result.

## Data Availability

STATA code used for data manipulation and analyses can be provided upon request.

## Abbreviations

ADO, Age, dyspnoea, and obstruction score; AUC, area-under-the-curve; BLISS, Birmingham Lung Improvement Studies; BMI, body mass index; CITL, Calibration-in-the-large; COPD, chronic obstructive pulmonary disease; CI, confidence interval; FEV1, forced expiratory volume in one second; FVC, forced vital capacity; mMRC, modified Medical Research Council.
